# Supplementary material for: Using patient biomarker time series to determine mortality risk in hospitalised COVID-19 patients: A comparative analysis across two New York hospitals
Source: PLoS One. 2022 Aug 18;17(8):e0272442. doi: 10.1371/journal.pone.0272442 (PMC9387798; doi:10.1371/journal.pone.0272442)
Supplement: Attachment — Submitted filename: covid_hospital_reviewer_comments.docx [file pone.0272442.s002.docx]

# *Response to reviewer comments*

*In what follows, we italicise our responses to the reviewers’ comments. We thank both reviewers for their thorough and useful reviews. We also thank the editor for their handling of the script.*

*Reviewer #1*

This manuscript evaluates a previously published Bayesian Markov model of hospitalized COVID-19 patient outcomes on data from 2 New York hospitals early in the pandemic. It is a reasonable approach to model evaluation in this context, which is nice to see, although I have a few concerns and this represents no more than an incremental contribution to the field.

*We are glad to hear that the reviewer finds our approach reasonable. In what follows, we seek to address their concerns on particular issues and are very appreciative for their useful comments.*

Major Concerns

1. In the post-admission data, is it the change in biomarker values from baseline that is informative or just having data from a later time point (which I would think is generally more predictive of a hospital outcome)? Comparing to a model that uses follow up covariate values would get at this.

*This is a very astute point and was an omission on our part during our initial submission. We have now repeated the analyses using the covariate values rather than the changes, and in the results, we now have a new section which compares the results from the two analyses (which we provide below). Overall, the predictive accuracies of the two models are virtually indistinguishable (Fig S13) meaning that we are unable to conclude whether it is the changes from a patient-specific baseline or the raw values of the biomarkers themselves that matter. There is, however, strong consistency in terms of the sign of the ORs across the two analyses: indicating a set of 13 biomarkers whose changes were associated with the same impact on mortality risk. To discuss these results, we include a new section of our results:*

*New results section title: “Relative changes in biomarker values and their raw values are similarly predictive of outcomes”*

*Body: “We tested the hypothesis that the biomarker values themselves opposed to their relative changes from a patient-specific baseline were most important in determining outcomes by fitting our multivariate Markov model using the biomarker values as covariates: we term this model the ``absolute values'' analysis to distinguish it from the ``relative changes'' analysis.*

*To do so, we compared the predictive power of the absolute values analysis with that of the relative changes one. In S13 Fig, we show the predictive accuracy in determining patients' outcomes for an independent hold-out hospital across the two analyses. This illustrates very similar accuracies across the two analyses, and that it is not possible for us to conclude whether the absolute biomarker values or the changes from baseline are most clinically relevant.*

*To explore whether the two analyses led to different conclusions about clinically relevant changes in biomarker values, we compared the ORs for mortality risk across the analyses. The ORs from the absolute values analysis were, like those from the relative changes analysis, very consistent across the hospitals (S14 Fig; rho=0.88, t_16=7.40, p<0.01; using posterior median estimates). We also compared the ORs across the two analyses (S15 Fig). The ORs produced across the two analyses were of the same sign for 15/18 biomarkers for SUNY and for 15/18 for Maimonides, and the ORs were strongly positively correlated for both hospitals (SUNY: rho=0.77, t_16=4.88, p<0.01; Maimonides: rho=0.94, t_16=11.4, p<0.01; both using posterior median estimates). Overall, there were six biomarkers whose increases were associated with increases in mortality risk across the two analyses and hospitals: BASO PCT, Creatine, LYM ABS, MCV, RDW and WBC; and a set of seven biomarkers whose increases led to reductions in risk: CO2, EOS PCT, Glucose Random, LYM PCT, MCH, MONO ABS and NEU ABS.”*

*We thank the reviewer for this extremely useful comment, which has led to a more comprehensive analysis.*

2. How would a naive black box machine learning algorithm fare at this prediction problem? This seems like a natural point of comparison, since these models (e.g., random forest, boosting, etc.) are so easy to use and widely available. To make a contribution, any prediction model must distinguish itself in some way from these readily available alternatives.

*This is an interesting point, and we agree that black box machine learning algorithms like those suggested would be an interesting point of comparison. There are, broadly, two qualities of a statistical model: its ability to be interrogated for explanation; and its ability to predict (Gait, 2010). And, dependent on the question being asked, one or other of these qualities may be more important.*

*Here, and in our previous paper (Momeni-Boroujeni et al., 2021), we sought to build a model that “quantified the mortality risk associated with both static (e.g. demographic and patient history variables) and dynamic factors (e.g. changes in biomarkers) throughout hospitalisation”. In doing so, we focused largely on whether our model could consistently explain the patterns in mortality across the two different settings. To make this clear, we have changed the corresponding sentence in the abstract to read,*

*“Our main focus was to quantify the mortality risk associated with both static (e.g. demographic and patient history variables) and dynamic factors (e.g. changes in biomarkers) throughout hospitalisation, by so doing, to explain the observed patterns of mortality.”*

*Similarly, we have changed the last sentence in our introduction to read,*

*“In doing so, we obtain an external validation of the model. More importantly, this allows us to appraise the use of dynamic biomarker measurements for determining patient mortality risk in hospitalised COVID-19 patients, which is our main contribution.”*

*In this paper, we aimed to determine whether the factors we previously found were associated with mortality risk (in State University of New York Downstate Medical Center) remained so in a new setting (Maimonides Medical Center). And Figs. 2, 3 & 4 demonstrate that there was remarkable consistency in the mortality risk found for the common biomarkers across the two hospitals. We also aimed to communicate these to a clinician audience, so we chose to use a model which allowed us to calculate odds ratios (Figs. 2 & 4), which are commonly used in the medical literature.*

*Our main focus was not to create and test a highly predictive prognostic model, and we caution against its use solely for predictive purposes in the final paragraph:*

*“Whilst our model performed well across the two cohorts examined, we caution against its use as a dynamic prognostic model in clinical settings. In order for it to be used as thus, any such model requires training and evaluation over a much larger sample size across multiple settings including the full set of factors implicated with risk. The ease of use in a clinical setting and effects on clinicians’ behaviour, comparison with existing prognostic models, cost-effectiveness and impact on patient health must also be assessed prior to the implementation of any prognostic model.”*

*We have also added a comment on the limitations of our model to the discussion and propose future work investigating an extension to the modelling framework along the lines suggested by the reviewer:*

*“By using a relatively simple model (which assumed a linear functional form on the log-odds scale), we focused on the ability of our model to explain not predict (Shimueli, 2010). Using this approach, it is possible that we missed important contributions from the interactions between factors, and future work could investigate the use of models such as Bayesian Additive Regression Trees (Chipman et al., 2010) which allow non-linear interactions between regressors.”*

*Shmueli, Galit. "To explain or to predict?" Statistical science 25.3 (2010): 289-310.*

*Momeni-Boroujeni, Amir, et al. "A dynamic bayesian model for identifying high-mortality risk in hospitalized COVID-19 patients." Infectious disease reports 13.1 (2021): 239-250.*

*Chipman, Hugh A., Edward I. George, and Robert E. McCulloch. "BART: Bayesian additive regression trees." The Annals of Applied Statistics 4.1 (2010): 266-298.*

3. I admit I am relatively ignorant of fitting models to MCHC, MCV and MCH. However, my understanding is that MCHC = MCH / MCV. Consequently, I consider it a little unusual to include all 3 as covariates in a model. Please explain. Could this be the reason that the MCHC and MCV estimates in lines 227-236 (and Fig S7A) seem so off?

*We are very grateful to the reviewer for this comment, and they are spot on — we reran our multivariate models without MCHC, and the ORs now look much more comparable between the two hospitals. Presumably, before, the high degree of multicollinearity between regressors induced by including MCHC, MCV and MCH was responsible for this instability.*

*As a result of this, we have now changed figure 4, which previously showed only a comparison between ORs for univariate models, to now show both univariate (panel A) and multivariate (panel B) estimates. (We have also deleted the old Figure S7 which compared the multivariate ORs.) We have also amended the text for this section (“Quantifying risk associated with dynamic biomarker measurements”) to now read:*

*“In Fig 4, we compare the ORs associated with daily mortality risk for each of the common biomarkers across the two hospitals as derived from the Markov model (described in Methods). Across the univariate and multivariate model estimates, there was strong positive correlation in the ORs between the hospitals (univariate: rho=0.84, t_16=6.52, p<0.01; multivariate: rho=0.65, t_16=3.45, p<0.01 ; in both cases, using posterior median estimates). With few exceptions the estimates agreed in terms of their ``sign'': for the univariate model, 15/18 tests had posterior median estimates where either both odds ratios were above one across the two hospitals or both were below one; for the multivariate model, the corresponding figure was 14/18 tests. A notable outlier was MCV, which was estimated to have a substantially stronger effect in the Maimonides cohort in the multivariate model, although this was not recapitulated in the univariate analysis suggesting caution interpreting this further.”*

*This has also resulted in minor changes to the reported accuracies, and the abstract now reads:*

*“The model accuracy in predicting patient outcome (death or discharge) was 72.3% (predicting SUNY; posterior median accuracy) and 71.3% (predicting Maimonides) respectively. Model sensitivity was higher for detecting patients who would go on to be discharged (78.7%) versus those who died (61.8%).”*

*There was also a minor change to Fig 5 as a result of this change to the analysis, and there is a new S11 Fig which shows minor changes from previous (which was S7). Otherwise, we have also updated the posterior predictive checks, and there are a few minor changes to statistics reported in the text to reflect the removal of MCHC from the model.*

Minor Concerns

1. Line 239: It might be worth making explicit mention in this paragraph that the evaluation uses out-of-sample predictions. That is a good approach, and telling the reader that here, up front, may alleviate some fears regarding suitability of the evaluation procedure that would not otherwise be allayed until later.

*We thank the reviewer for their suggestion and agree. We have now changed the first sentence of this section to read:*

*“Across the different regressor sets, out-of-sample predictive accuracy was consistent across the hospitals (Fig 5A).”*

2. Line 247: Please correct the grammatical error in “groups patients”.

*Thank you, we have changed this.*

3. Line 248-251: I believe this confusion matrix uses in-sample predictions. Some type of out-of-sample estimator would be more compelling, e.g., cross-validation.

*This was actually formed using out-of-sample predictions for both between-hospital comparisons: when training the model using data from Maimonides to determine outcomes in SUNY and vice versa. Our “pooling” was only to group together the prediction results: the number of times we predicted discharge when death actually occurred, the number of times when we predicted discharge when this is what occurred, and so on. We recognise that this is a potential point of confusion, and we have amended the text to this section to hopefully mitigate against this:*

*“To do so, we pooled predictions across both independent hospital testing sets: note, that in both cases, these predictions were formed using out-of-sample testing sets.”*

4. Line 270-275: It would be nice to show the entire set of variable importance scores in the supplemental material.

*Thank you for this suggestion. We now include these as Fig S12.*

5. Supplemental Figure 7: The caption refers to a “dashed black line”, when the line is solid.

*We have now removed this figure following the discussion above around the non-inclusion of MCHC in the model.*

6. Supplemental Figure 7: Panel b doesn’t just zoom in, but refits the blue line on a reduced set of features (which the text notes, but the caption is misleading)

*We have now removed this figure following the discussion above around the non-inclusion of MCHC in the model.*

## *Reviewer #2*

This article follows up on an earlier study by the authors that used time-varying covariates to model mortality risk in COVID-19 patients hospitalized in a New York hospital.

The focus of the article is on results obtained using data from an additional hospital, as well using the data from each of the two hospitals for the purposes of external validation of the model trained using data from the other. These are both useful contributions. As such, the article is well-written and clearly articulates its goals, findings, and limitations. The methodology appears to be sound.

*We thank the reviewer for their summation and are pleased that they agree that the article is a useful contribution.*

However, I do feel that a fundamentally important quantity has not been clearly defined, either in this or the original article. Specifically, what exactly is the 'prediction' made by the dynamic model?

*This is a key point and was an omission in our submission. Our predictive accuracy was the fraction of time that the patient’s ultimate outcome – death or discharge – was predicted correctly by the model. We now make this clear with the Methods:*

*“Next, we assessed whether the sets of factors considered in this paper can be used to predict patient outcomes that generalise across both hospitals. To do so, we fitted the Markov model to data from each hospital in turn, then used it to predict patient outcomes (i.e. whether the patient ultimately died in hospital or was discharged) in the other held-out hospital.”*

Fig 5A compares the internal and external accuracies of the 'prediction' made by four models. Here, the dynamic 'post-admission' model shows a clear improvement (at around 72% accuracy) over the next best 'admission' model (at around 60%). The prediction made by the admission model is unambiguous: for any particular patient, it is based on the patient covariates at the time of admission.

However, for the dynamic model, a prediction can be made for each patient for every day that the patient stays in the hospital. These will depend on the parameters of the trained model as well as the current values of the patient's biomarkers. Which of these several possible predictions have been used to arrive at the prediction accuracy for the post-admission model in Fig 5A? At a minimum, I think this needs to be clearly stated, especially as the unavailability of the raw data makes it impossible to reproduce the analysis.

If for example, the prediction used is the one on the last day of stay (before death or discharge), then the improvement in accuracy may not be as impressive. In that case, a more appropriate quantity to compare could be the predictions from the admission model using the corresponding day's biomarker values (instead of the values at admission).

*The prediction is not that on the last day of stay; rather it is the model’s simulations of what will happen to each patient ultimately – whether they are discharged or die. To make it clear that we do not consider the timing of events, we include the following in Methods:*

*“In this analysis, we did not consider the time taken for death or discharge to occur, and future work could consider also these outcomes (although our previous work has demonstrated that predicting timings is likely difficult; Momeni-Boroujeni et al. (2021)).*

*We have now obtained permission to make the raw data for this study available, and we publish it through a Zenodo repository: https://doi.org/10.5281/zenodo.6771834.*

*Momeni-Boroujeni, Amir, et al. "A dynamic Bayesian model for identifying high-mortality risk in hospitalized COVID-19 patients." Infectious disease reports 13.1 (2021): 239-250.*

Also, regardless of the definition used, I think it may be useful to quantify and visualize the progression of the daily prediction accuracy over hospital stay. The varying length of hospital stay makes this not straightforward, but perhaps the authors could consider a plot of accuracy of prediction made with covariates available k days before actual death or discharge, with k varying from 1 to 10 or so.

*We thank the reviewer for these suggestions. We attempted a range of different visualisations showing the temporal variation in predictive accuracy, but, generally, the value of these was unclear owing to the relatively few patients with long stays in hospital.*

*Below, we provide a plot below which shows the daily predictive accuracy for patients who were either discharged or died as a function of hospital stay (this was for a model using the dynamic biomarker changes from baseline fitted to the SUNY data and exploring the predictive accuracy on independent data from Maimonides hospital). This figure suggests that, overall, predictive accuracy increased over duration of stay for those who ultimately went on to be discharged whereas the trends over time were more ambiguous for those who eventually died. But, these trends were quite sensitive to the relatively few patients who had longer hospital stays, and there was considerable heterogeneity in these patterns. As such, we chose not to include this graph as part of our resubmission.*

I noticed a couple of minor typos around lines 41-42 (grammar) and line 241 (the word 'accuracy' is missing).

*Thank you. We have restructured the end of the introduction (lines 41-42) to read:*

*“In doing so, we obtain an external validation of the model. More importantly, this allows us to appraise the use of dynamic biomarker measurements for determining patient mortality risk in hospitalised COVID-19 patients, which is our main contribution.”*

*We have also added in the word “accuracy” on line 241.*
